# Supplementary material for: Kronos scRT: a uniform framework for single-cell replication timing analysis
Source: Nat Commun. 2022 Apr 28;13:2329. doi: 10.1038/s41467-022-30043-x (PMC9050662; doi:10.1038/s41467-022-30043-x)
Supplement: Supplementary file 1 — Supplementary Information [file 41467_2022_30043_MOESM1_ESM.pdf]

SUPPLEMENTARY INFORMATION

Kronos scRT: a uniform framework for single-cell replication timing analysis

Gnan et al.

Supplementary Table 1. Summary of the scRT data analysed in the current study.

Species	Cell lines	Cell enrichment	Method	Number of cells				Ref (raw data)
				Whole sample	Passed filters*	G1/G2-phase	S-phase	
Mouse	mESC	G1 sorted & mid-S sorted	scWGA	67	67	13	54	Ref[22]
	mNE-7d	G1 sorted & mid-S sorted	scWGA	45	45	3	42	Ref[22]
	mESC 2i	Cycling cells	scHi-C	1440	641	312	329	Ref[31]
	mESC Serum	Cycling cells	scHi-C	437	206	76	130	Ref[31]
Human	MCF7	Cycling cells	10x scCNV	447	368	286	82	This study
	MCF7	S-phase enriched	10x scCNV	2321	1777	424	1353	This study
	HeLa	S-phase enriched	10x scCNV	752	514	255	259	This study
	Jeff	S-phase enriched	10x scCNV	1455	1106	146	960	This study

*Based on the simulation results shown in Fig. 1d and Supplementary Fig. 1b,c, stringent thresholds (Supplementary Table 2) were used in the present study to select high-quality cells in order to avoid confounding technical noise for cell-to-cell variations.

Supplementary Table 2. Summary of the parameters used in the analysis.

Cell line	Run	Read type	Ploidy limits	G1/G2-phase threshold	S-phase threshold	Adj. 1st part S-phase	Adj. 2nd part S-phase	Adj. type	Reads per Mb threshold
HeLa	1	PE	[2.0, 8.0]	0.90	0.85	0.950	0.540	Auto	117
	2	PE	[2.0, 8.0]	0.90	0.60	0.950	0.537	Auto	117
Jeff	1	PE	[1.3, 4.3]	0.80	0.75	0.950	0.550	Auto	117
	2	PE	[1.3, 4.3]	0.60	0.50	0.951	0.524	Auto	117
Unsorted MCF7	single	PE	[2.5, 8.0]	Automatic		0.950	0.550	Auto	117
S-phase enriched MCF7	single	PE	[2.5, 8.0]	0.70	0.75	0.950	0.550	Auto	117
mESC 2i	--	SE	[1.3, 4.3]	1.00	0.90	0.950	0.550	Auto	138
mESC Serum	--	SE	[1.3, 4.3]	1.00	0.90	1.000	0.500	Auto	138
mESC scRT	--	SE	~2.0	WhoIsWho		0.900	0.550	Manual	163
NE-7d	--	SE	~2.0	WhoIsWho		0.900	0.550	Manual	163

a

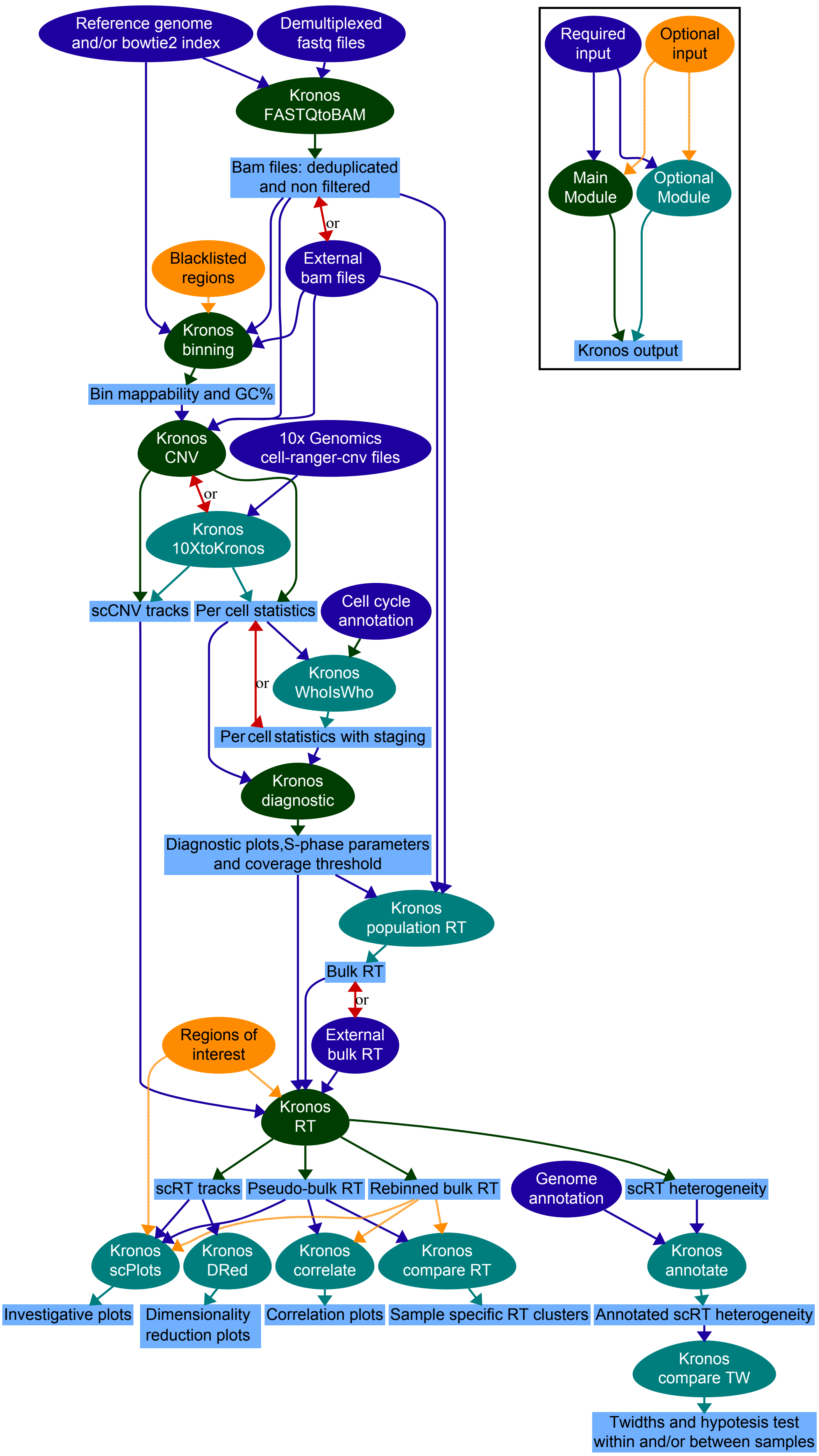

Reference genome and/or bowtie2 index
Demultiplexed fastq files
Kronos FASTQtoBAM
Bam files: deduplicated and non filtered
or
External bam files
Blacklisted regions
Kronos binning
Bin mappability and GC%
Kronos CNV
10x Genomics cell-ranger-cnv files
or
Kronos 10XtoKronos
scCNV tracks
Per cell statistics
Cell cycle annotation
or
Kronos WhoIsWho
Per cell statistics with staging
Kronos diagnostic
Diagnostic plots,S-phase parameters and coverage threshold
Kronos population RT
Bulk RT
or
External bulk RT
Regions of interest
Kronos RT
scRT tracks
Pseudo-bulk RT
Rebinned bulk RT
Genome annotation
scRT heterogeneity
Kronos scPlots
Kronos DRed
Kronos correlate
Kronos compare RT
Kronos annotate
Investigative plots
Dimensionality reduction plots
Correlation plots
Sample specific RT clusters
Annotated scRT heterogeneity
Kronos compare TW
Twidths and hypotesis test within and/or between samples
Required input
Optional input
Main Module
Optional Module
Kronos output

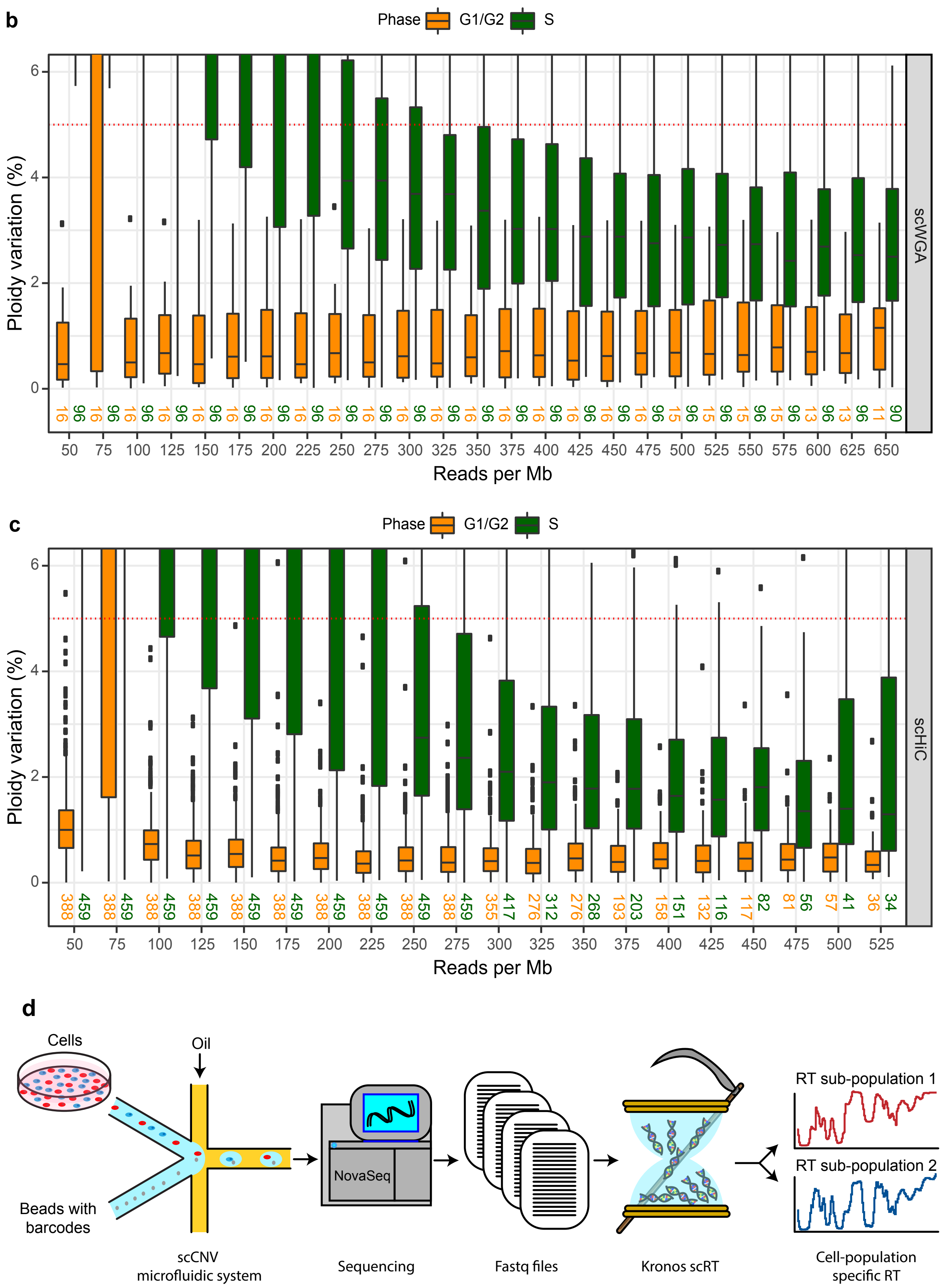

b
Phase
G1/G2
S
Ploidy variation (%)
Reads per Mb
scWGA
c
Phase
G1/G2
S
Ploidy variation (%)
Reads per Mb
scHiC
d
Cells
Oil
Beads with barcodes
scCNV microfluidic system
NovaSeq
Sequencing
Fastq files
Kronos scRT
RT sub-population 1
RT sub-population 2
Cell-population specific RT

Supplementary Figure 1. Kronos scRT framework. a Complete Kronos scRT pipeline with all the developed modules. Input files, main modules, and optional modules are shown in green, dark blue, and light blue, respectively. **b-c** Reads down-sampling as in Fig. 1d for scWGA and scHi-C data obtained by Takahashi et al 2019[22] and Nagano et al. 2017[31], respectively. The numbers of cells (*n*) used for each down-sampling are reported below each boxplot, cells are analysed over one experiment. In the boxplots, bounds of box: 25th and 75th percentiles; centre line: median; lower (upper) whisker: lower (upper) bound - (+) 1.5 x Interquartile range (IQR). **d** Schematic representation of a typical analysis starting from the experimental set-up to the final results for a mixed cell population.

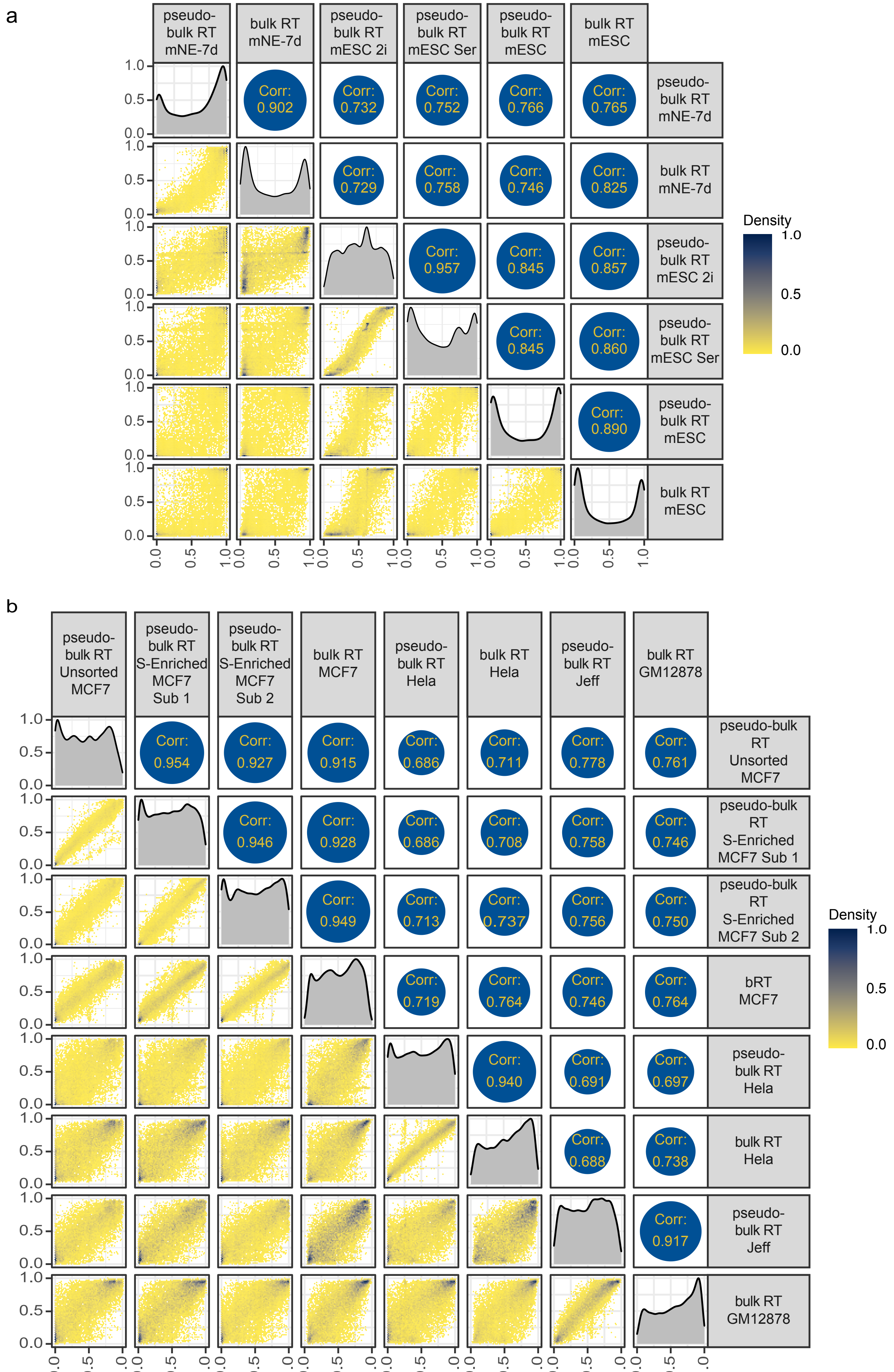
a
pseudo-bulk RT mNE-7d
bulk RT mNE-7d
pseudo-bulk RT mESC 2i
pseudo-bulk RT mESC Ser
pseudo-bulk RT mESC
bulk RT mESC
Corr: 0.902
Corr: 0.732
Corr: 0.752
Corr: 0.766
Corr: 0.765
Corr: 0.729
Corr: 0.758
Corr: 0.746
Corr: 0.825
Corr: 0.957
Corr: 0.845
Corr: 0.857
Corr: 0.845
Corr: 0.860
Corr: 0.890
Density
1.0
0.5
0.0
b
pseudo-bulk RT Unsorted MCF7
pseudo-bulk RT S-Enriched MCF7 Sub 1
pseudo-bulk RT S-Enriched MCF7 Sub 2
bulk RT MCF7
pseudo-bulk RT Hela
bulk RT Hela
pseudo-bulk RT Jeff
bulk RT GM12878
bRT MCF7
Corr: 0.954
Corr: 0.927
Corr: 0.915
Corr: 0.686
Corr: 0.711
Corr: 0.778
Corr: 0.761
Corr: 0.946
Corr: 0.928
Corr: 0.686
Corr: 0.708
Corr: 0.758
Corr: 0.746
Corr: 0.949
Corr: 0.713
Corr: 0.737
Corr: 0.756
Corr: 0.750
Corr: 0.719
Corr: 0.764
Corr: 0.746
Corr: 0.764
Corr: 0.940
Corr: 0.691
Corr: 0.697
Corr: 0.688
Corr: 0.738
Corr: 0.917
Density
1.0
0.5
0.0

Supplementary Figure 2. Bulk and pseudo-bulk RT comparison. a-b Circles: pair-wise Spearman correlations of the bulk and pseudo-bulk RT profiles in the indicated mouse (a) and human (b) cell lines. The histograms represent the RT distribution of each sample, and the 2D density plots show the pair-wise comparisons between samples (colour code is shown on the right).

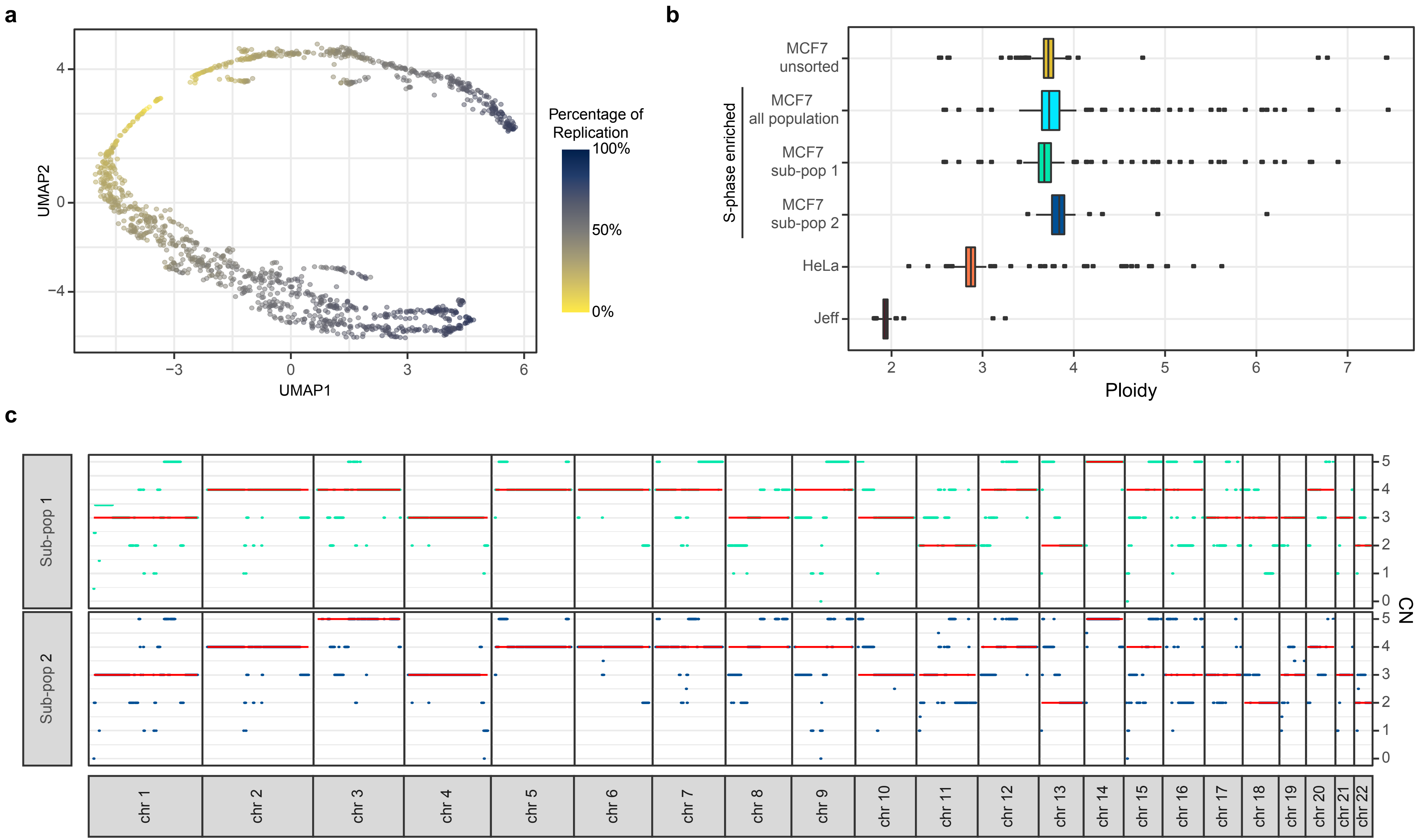

a
UMAP2
UMAP1
4
0
−4
−3
0
3
6
Percentage of Replication
100%
50%
0%
b
MCF7 unsorted
MCF7 all population
MCF7 sub-pop 1
MCF7 sub-pop 2
S-phase enriched
HeLa
Jeff
Ploidy
2
3
4
5
6
7
c
Sub-pop 1
Sub-pop 2
CN
5
4
3
2
1
0
chr 1
chr 2
chr 3
chr 4
chr 5
chr 6
chr 7
chr 8
chr 9
chr 10
chr 11
chr 12
chr 13
chr 14
chr 15
chr 16
chr 17
chr 18
chr 19
chr 20
chr 21
chr 22

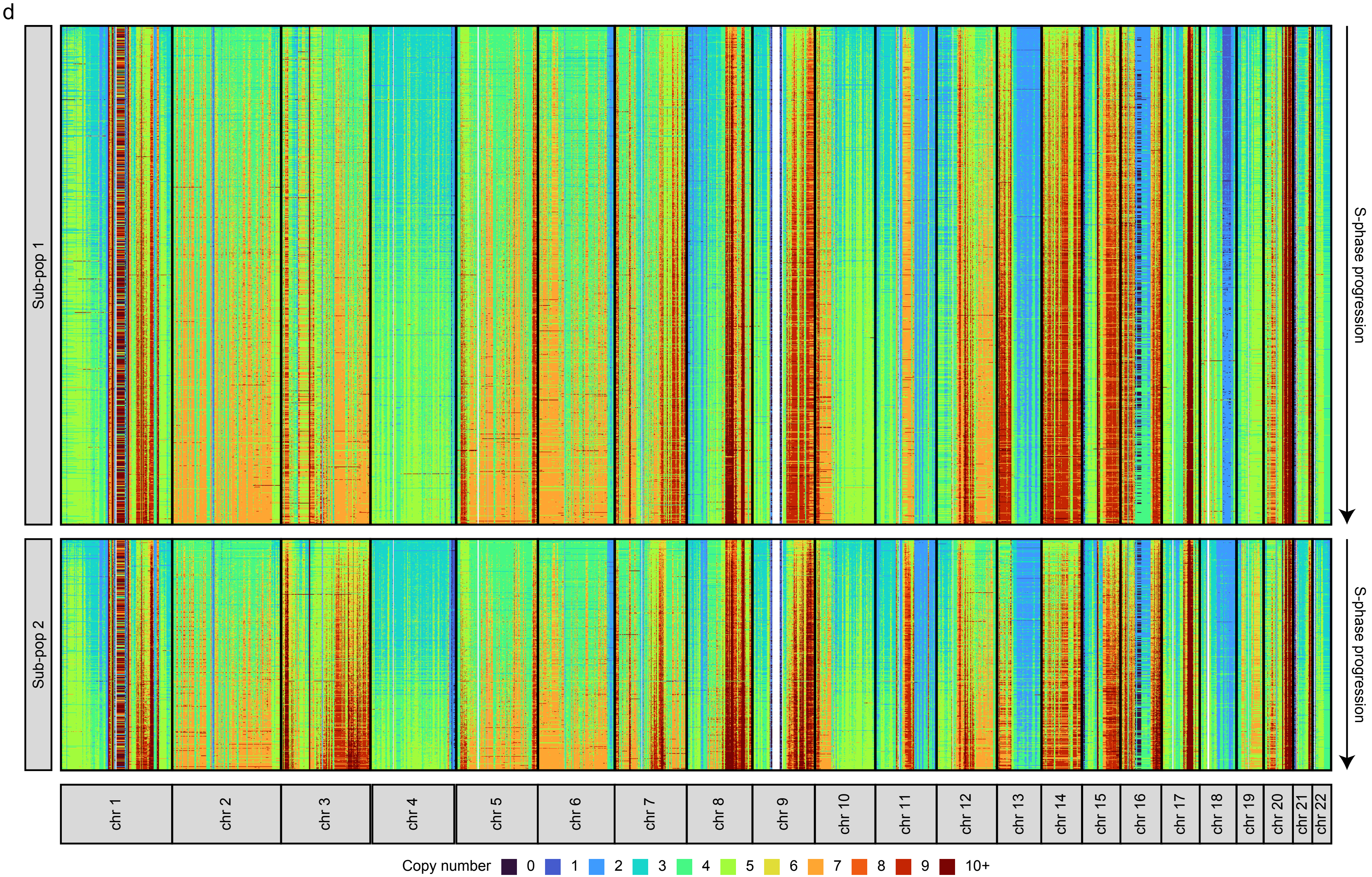

d
Sub-pop 1
Sub-pop 2
S-phase progression
S-phase progression
chr 1
chr 2
chr 3
chr 4
chr 5
chr 6
chr 7
chr 8
chr 9
chr 10
chr 11
chr 12
chr 13
chr 14
chr 15
chr 16
chr 17
chr 18
chr 19
chr 20
chr 21
chr 22
Copy number 0 1 2 3 4 5 6 7 8 9 10+

Supplementary Figure 3. The MCF7 cell culture contains two sub-populations. a Dimensionality reduction analysis of scRT profiles. Each dot represents a single cell that is coloured in function of its percentage of genome replication. **b** Boxplots showing the mean ploidy of each cell calculated by Kronos scRT using the G1/G2-phase population. In the boxplots, bounds of box: 25th and 75th percentiles; centre line: median; lower (upper) whisker: lower (upper) bound - (+) 1.5 x IQR. HeLa: n=255 cells over 2 independent experiments; Jeff: n=146 cells over 2 independent experiments; unsorted MCF7: n=286 cells over one experiment; S-phase enriched MCF7 cells: n=424 cells (with n=271 cells of sub-pop 1 and n=153 cells of sub-pop 2) over one experiment. **c** Median copy number (CN) along autosomal chromosomes in the G1/G2-phase MCF7 cell sub-population 1 (aqua) and sub-population 2 (blue) (bin size = 1 Mb). Red lines represent the median CN of the whole chromosome. **d** CN along autosomes detected in the S-phase MCF7 cells. Cells were grouped based on the corresponding G1/G2-phase shown in Fig. 4a. Cells were sorted based on S-phase progression.

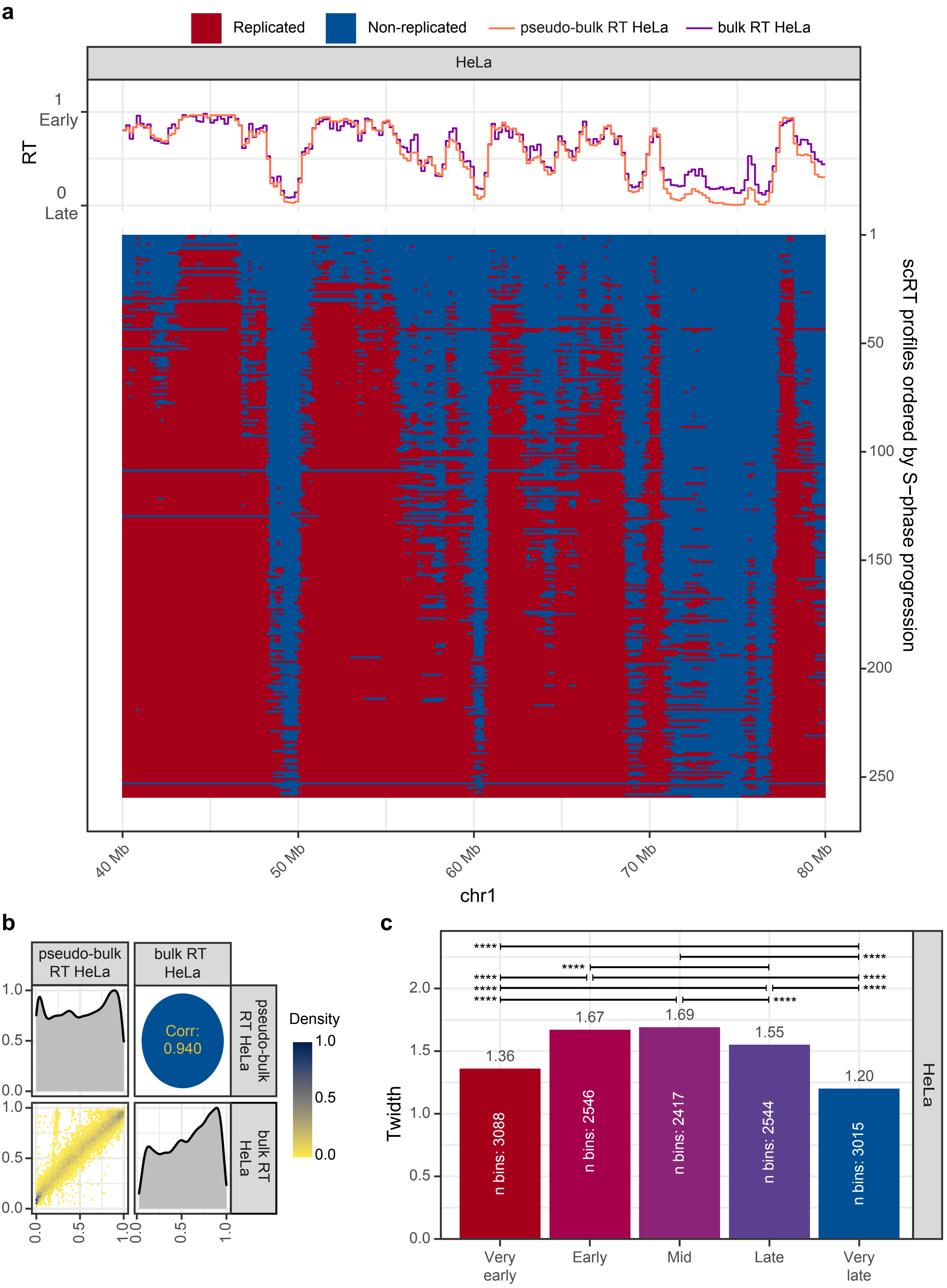
a
Replicated
Non-replicated
pseudo-bulk RT HeLa
bulk RT HeLa
HeLa
RT
1
Early
0
Late
scRT profiles ordered by S−phase progression
1
50
100
150
200
250
40 Mb
50 Mb
60 Mb
70 Mb
80 Mb
chr1
b
pseudo-bulk RT HeLa
bulk RT HeLa
Corr: 0.940
pseudo-bulk RT HeLa
bulk RT HeLa
Density
1.0
0.5
0.0
c
Twidth
****
1.36
1.67
1.69
1.55
1.20
n bins: 3088
n bins: 2546
n bins: 2417
n bins: 2544
n bins: 3015
Very early
Early
Mid
Late
Very late
HeLa

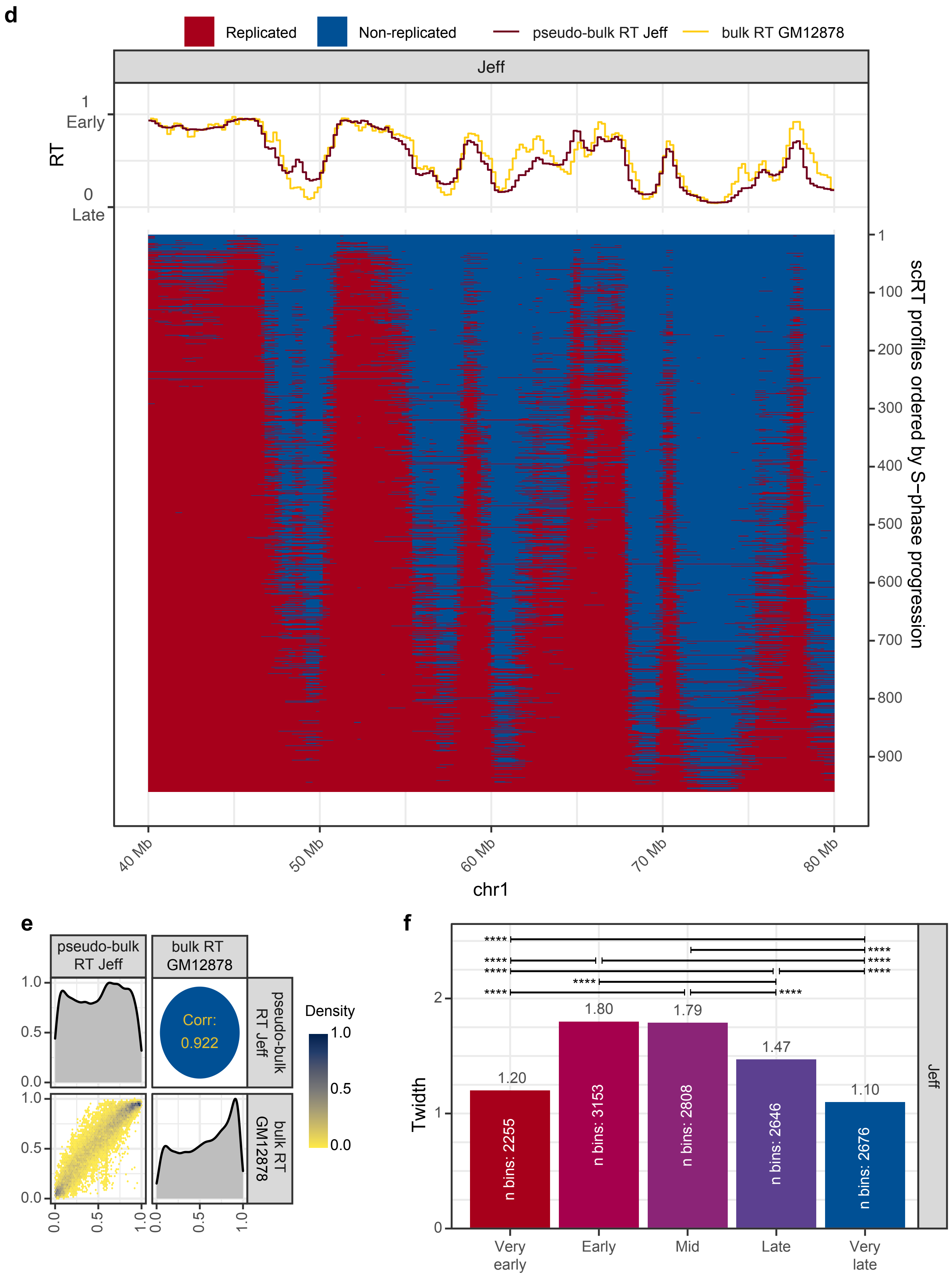

d
Replicated
Non-replicated
pseudo-bulk RT Jeff
bulk RT GM12878
Jeff
RT
1
Early
0
Late
scRT profiles ordered by S−phase progression
1
100
200
300
400
500
600
700
800
900
40 Mb
50 Mb
60 Mb
70 Mb
80 Mb
chr1
e
pseudo-bulk RT Jeff
bulk RT GM12878
pseudo-bulk RT Jeff
bulk RT GM12878
Corr:
0.922
Density
1.0
0.5
0.0
1.0
0.5
0.0
0.0
0.5
1.0
f
Twidth
Jeff
****
1.20
1.80
1.79
1.47
1.10
n bins: 2255
n bins: 3153
n bins: 2808
n bins: 2646
n bins: 2676
Very early
Early
Mid
Late
Very late
0
1
2

Supplementary Figure 4. scRT from S-phase enriched human cells (extension of Fig. 5 for HeLa and Jeff cells). a,d The scRT profiles of S-phase enriched HeLa (a) and Jeff (d) cells along a representative region. Top: pseudo-bulk RT and bulk RT profiles of the corresponding cell line. Bottom: scRT profiles ordered from top to bottom in function of the genome replication percentage of each cell. **b,e** Pairwise comparison of pseudo-bulk RT and bulk RT in HeLa (b) and Jeff cells (e). Same as in Supplementary Fig. 2. **c,f** T_{width} values calculated for the indicated five RT categories based on the pseudo-bulk RT values in HeLa (c) and Jeff (f) cells. Categories were selected as in Fig. 3d. P-values were calculated using the Kronos scRT Compare TW module (Methods); * < 0.05, ** < 10^{-2}, *** < 10^{-3}, **** < 10^{-4}.

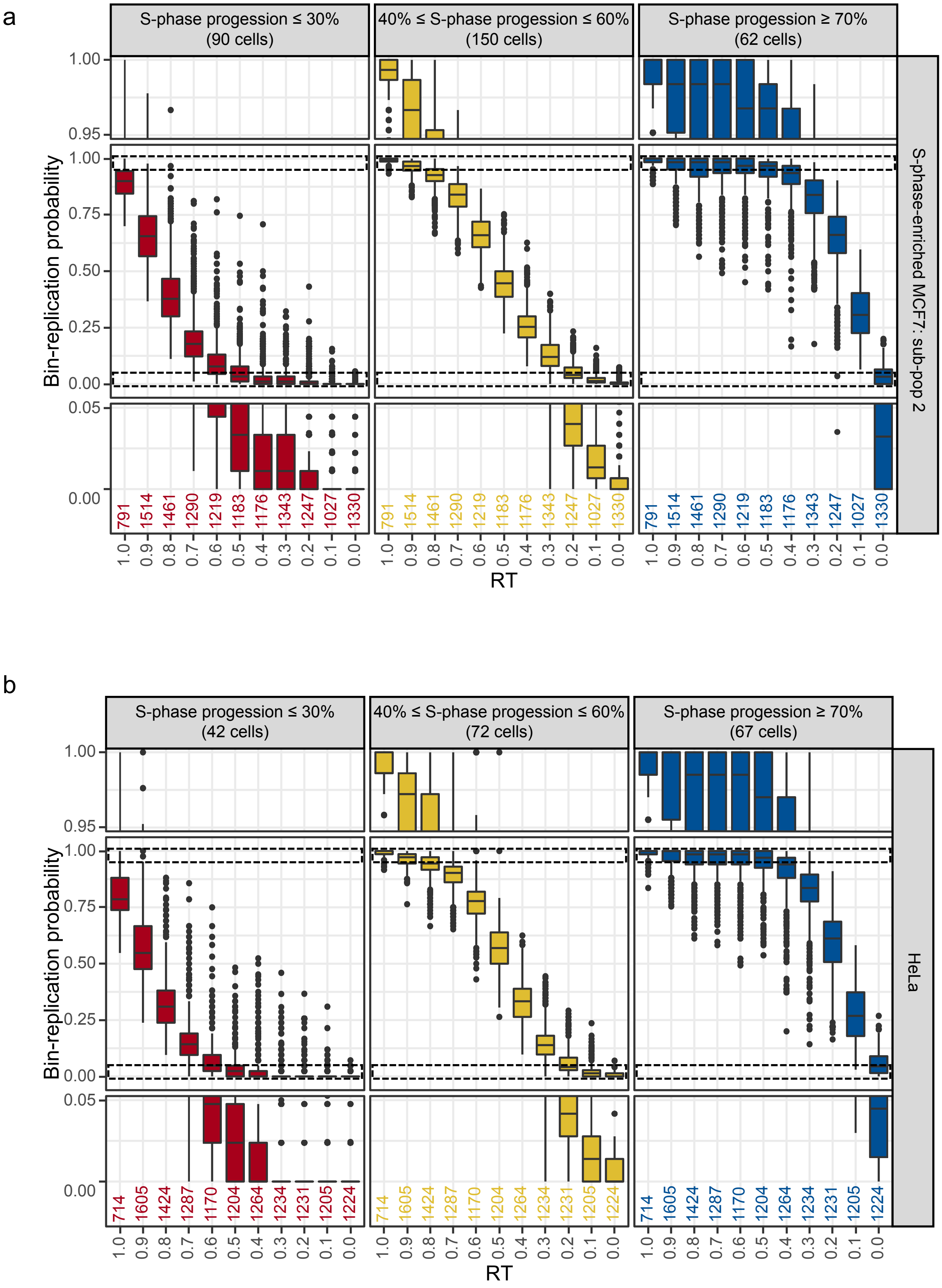

a
S-phase progession ≤ 30%
(90 cells)
40% ≤ S-phase progession ≤ 60%
(150 cells)
S-phase progession ≥ 70%
(62 cells)
S-phase-enriched MCF7: sub-pop 2
Bin-replication probability
1.00
0.95
0.75
0.50
0.25
0.00
0.05
791 1514 1461 1290 1219 1183 1176 1343 1247 1027 1330
1.0 0.9 0.8 0.7 0.6 0.5 0.4 0.3 0.2 0.1 0.0
RT
b
S-phase progession ≤ 30%
(42 cells)
40% ≤ S-phase progession ≤ 60%
(72 cells)
S-phase progession ≥ 70%
(67 cells)
HeLa
Bin-replication probability
714 1605 1424 1287 1170 1204 1264 1234 1231 1205 1224
1.0 0.9 0.8 0.7 0.6 0.5 0.4 0.3 0.2 0.1 0.0
RT

c

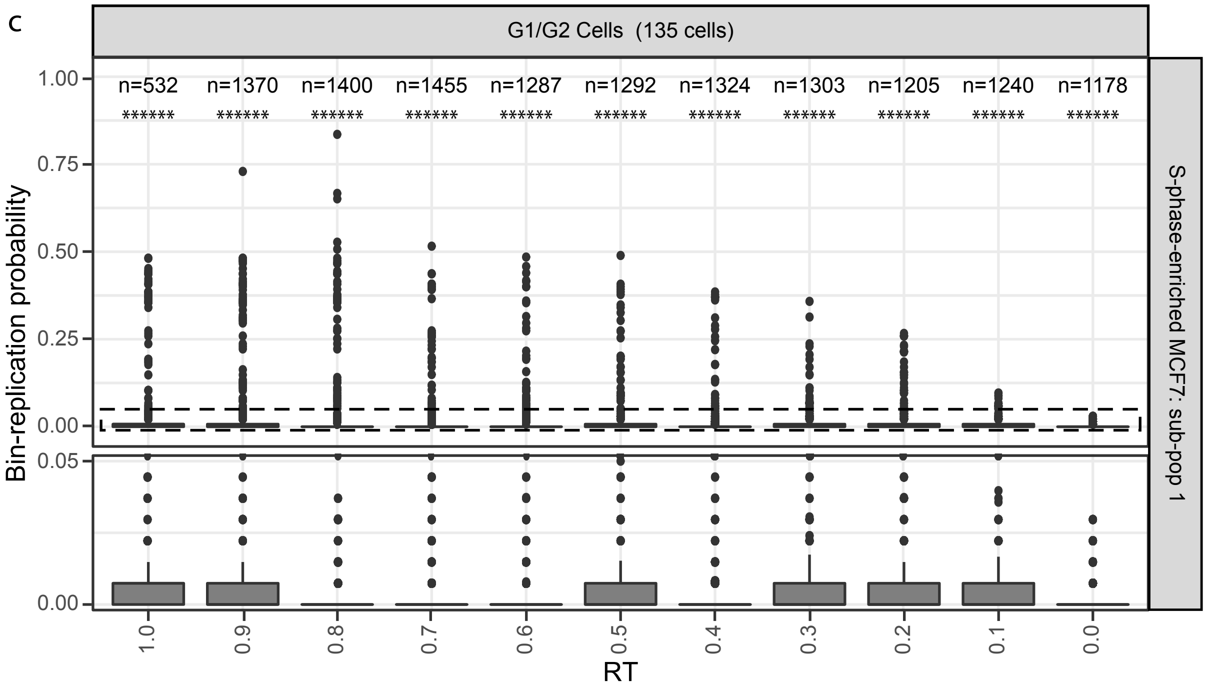
G1/G2 Cells (135 cells)
S-phase-enriched MCF7: sub-pop 1
Bin-replication probability
1.00
0.75
0.50
0.25
0.00
0.05
0.00
n=532
n=1370
n=1400
n=1455
n=1287
n=1292
n=1324
n=1303
n=1205
n=1240
n=1178
******
1.0
0.9
0.8
0.7
0.6
0.5
0.4
0.3
0.2
0.1
0.0
RT

d

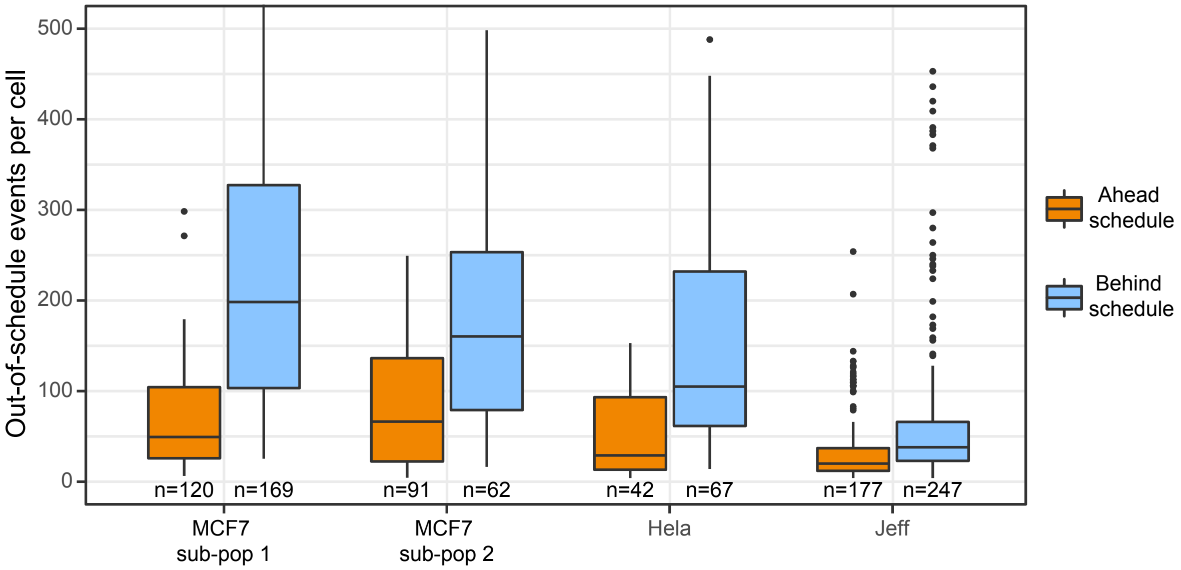
Out-of-schedule events per cell
500
400
300
200
100
0
n=120
n=169
n=91
n=62
n=42
n=67
n=177
n=247
MCF7
sub-pop 1
MCF7
sub-pop 2
Hela
Jeff
Ahead
schedule
Behind
schedule

e

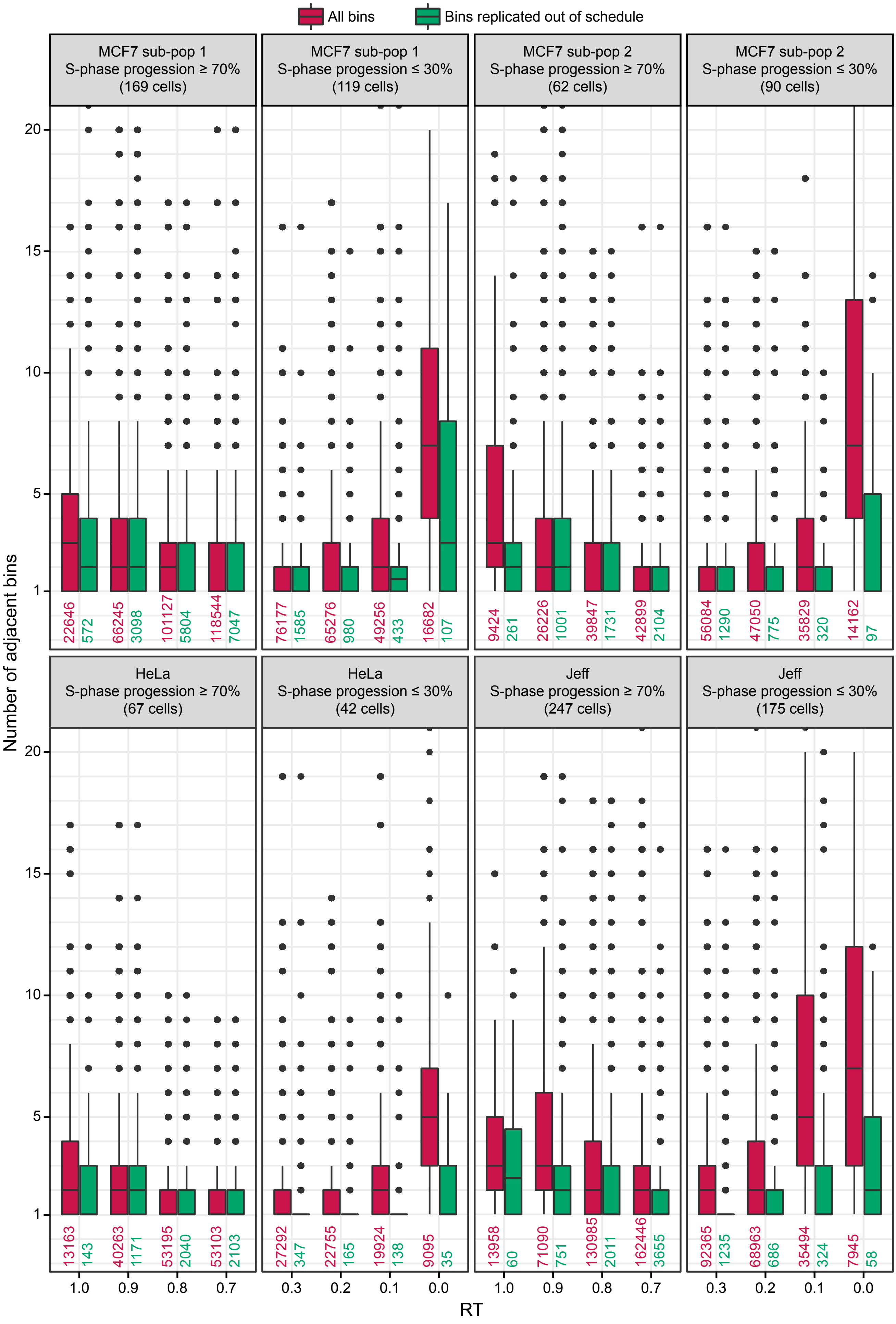

All bins
Bins replicated out of schedule
MCF7 sub-pop 1
S-phase progression ≥ 70%
(169 cells)
MCF7 sub-pop 1
S-phase progression ≤ 30%
(119 cells)
MCF7 sub-pop 2
S-phase progression ≥ 70%
(62 cells)
MCF7 sub-pop 2
S-phase progression ≤ 30%
(90 cells)
HeLa
S-phase progression ≥ 70%
(67 cells)
HeLa
S-phase progression ≤ 30%
(42 cells)
Jeff
S-phase progression ≥ 70%
(247 cells)
Jeff
S-phase progression ≤ 30%
(175 cells)
Number of adjacent bins
RT
22646 572 66245 3098 101127 5804 118544 7047
76177 1585 65276 980 49256 433 16682 107
9424 261 26226 1001 39847 1731 42899 2104
56084 1290 47050 775 35829 320 14162 97
13163 143 40263 1171 53195 2040 53103 2103
27292 347 22755 165 19924 138 9095 35
13958 60 71090 751 130985 2011 162446 3655
92365 1235 68963 686 35494 324 7945 58

f

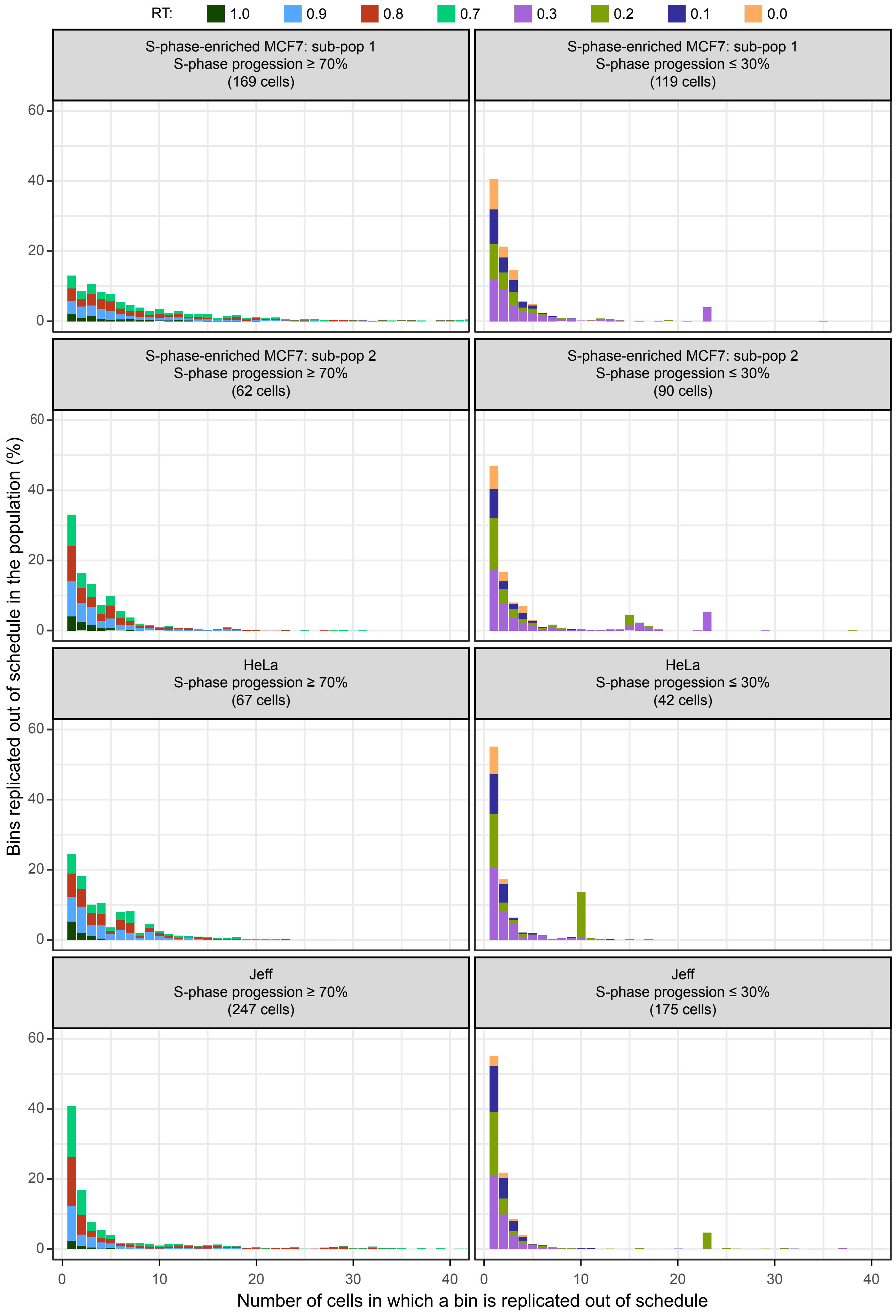

RT: 1.0 0.9 0.8 0.7 0.3 0.2 0.1 0.0
S-phase-enriched MCF7: sub-pop 1
S-phase progession ≥ 70%
(169 cells)
S-phase-enriched MCF7: sub-pop 1
S-phase progession ≤ 30%
(119 cells)
S-phase-enriched MCF7: sub-pop 2
S-phase progession ≥ 70%
(62 cells)
S-phase-enriched MCF7: sub-pop 2
S-phase progession ≤ 30%
(90 cells)
HeLa
S-phase progession ≥ 70%
(67 cells)
HeLa
S-phase progession ≤ 30%
(42 cells)
Jeff
S-phase progession ≥ 70%
(247 cells)
Jeff
S-phase progession ≤ 30%
(175 cells)
0
20
40
60
0
10
20
30
40
Bins replicated out of schedule in the population (%)
Number of cells in which a bin is replicated out of schedule

g

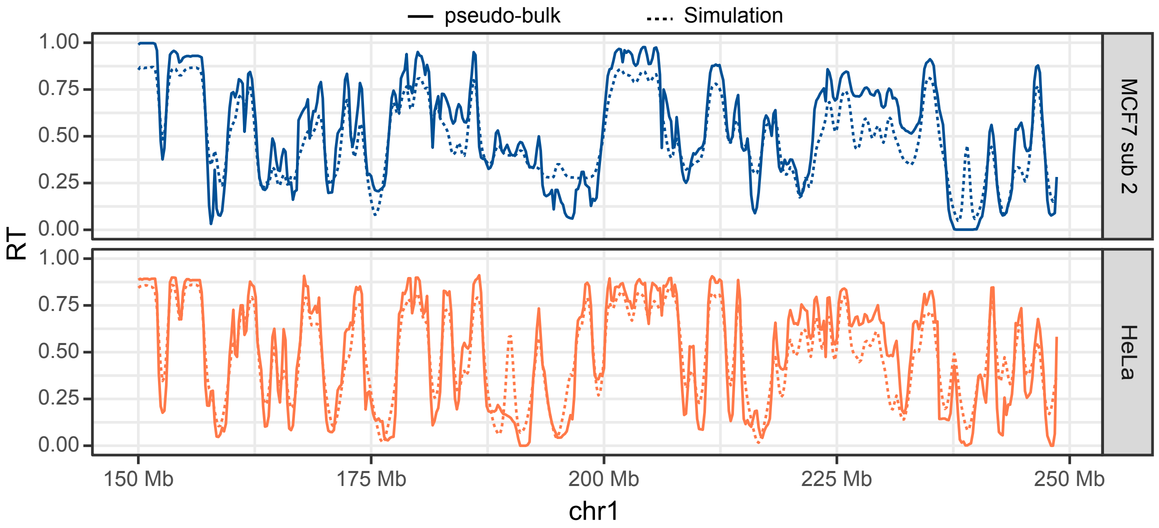

pseudo-bulk
Simulation
RT
1.00
0.75
0.50
0.25
0.00
MCF7 sub 2
HeLa
150 Mb
175 Mb
200 Mb
225 Mb
250 Mb
chr1

h

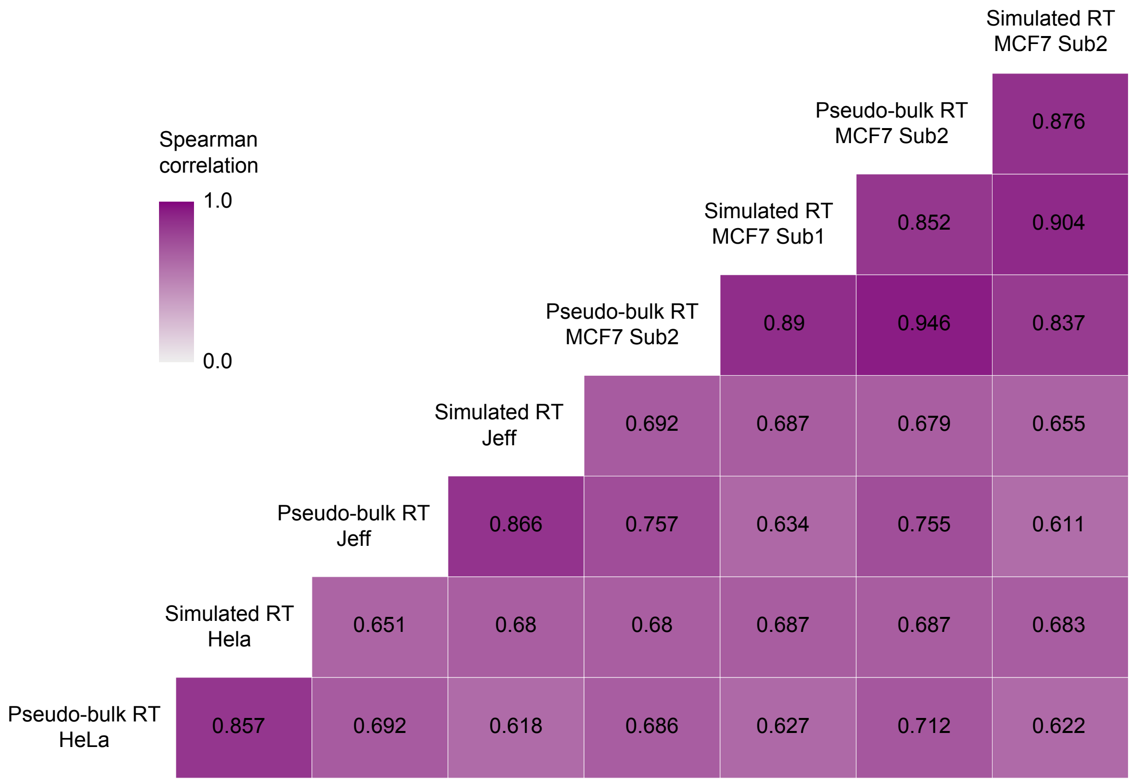

Spearman correlation
1.0
0.0
Simulated RT MCF7 Sub2
Pseudo-bulk RT MCF7 Sub2
Simulated RT MCF7 Sub1
Pseudo-bulk RT MCF7 Sub2
Simulated RT Jeff
Pseudo-bulk RT Jeff
Simulated RT Hela
Pseudo-bulk RT HeLa
0.876
0.852
0.904
0.89
0.946
0.837
0.692
0.687
0.679
0.655
0.866
0.757
0.634
0.755
0.611
0.651
0.68
0.68
0.687
0.687
0.683
0.857
0.692
0.618
0.686
0.627
0.712
0.622

Supplementary Figure 5. scRT data support a stochastic model of replication (extension of Fig. 6). a-b Boxplots showing the replication probability relative to its pseudo-bulk RT for the S-phase-enriched MCF7 cell sub-population 2 (a) and HeLa (b) cells, same as Fig. 6a-b. **c** Boxplots showing the replication probability relative to the pseudo-bulk RT (calculated in the G1/G2 cells of MCF7 cell sub-population 1). To guarantee only containing cells out of S phase, only cells with a mean ploidy included in the interquartile of the population were selected. P-values were calculated using the one-sided paired Wilcoxon test with the alternative hypothesis that replication variability is lower in the G1/G2-phase population than in early S-phase population (S-phase progression ≤30%, Fig 6A) (****** $p < 10^{-6}$). **d** Boxplots showing the number of out-of-schedule events per cell. Orange, bins replicated ahead of schedule (bins replicated within regions with a pseudo-bulk RT ≤0.3 in cells that went through the first 30% of S-phase). Blue, bins replicated behind schedule (non-replicated bins in regions with a pseudo-bulk RT ≥0.7 in cells that have gone through at least 70% of S-phase). **e** Boxplots showing the number of adjacent genomic bins in function of the pseudo-bulk RT. Magenta, all genomic bins with the corresponding pseudo-bulk RT. Green, only bins that replicated out-of-schedule (same definition as in d) in a cell. **f** Histograms showing the proportion of bins that are replicated out-of-schedule (same definition as in d) in function of the number of cells sharing the same event. **g** Comparison of the pseudo-bulk RT (solid line) and simulated RT (dashed line) profiles of MCF7 cell sub-population 2 and HeLa cells, same as Fig. 6c. **h** Pairwise Spearman correlations of the pseudo-bulk RT and simulated RT profiles for all the samples used in this study. In all boxplots, bounds of box: 25th and 75th percentiles; centre line: median; lower(upper) whisker: lower(upper) bound -(+) 1.5 x IQR; and the numbers of genomic bins (Fig. S5a-c), numbers of total adjacent bins (Fig. 5e), or numbers of cells (Fig. S5d) are reported for each corresponding boxplot.

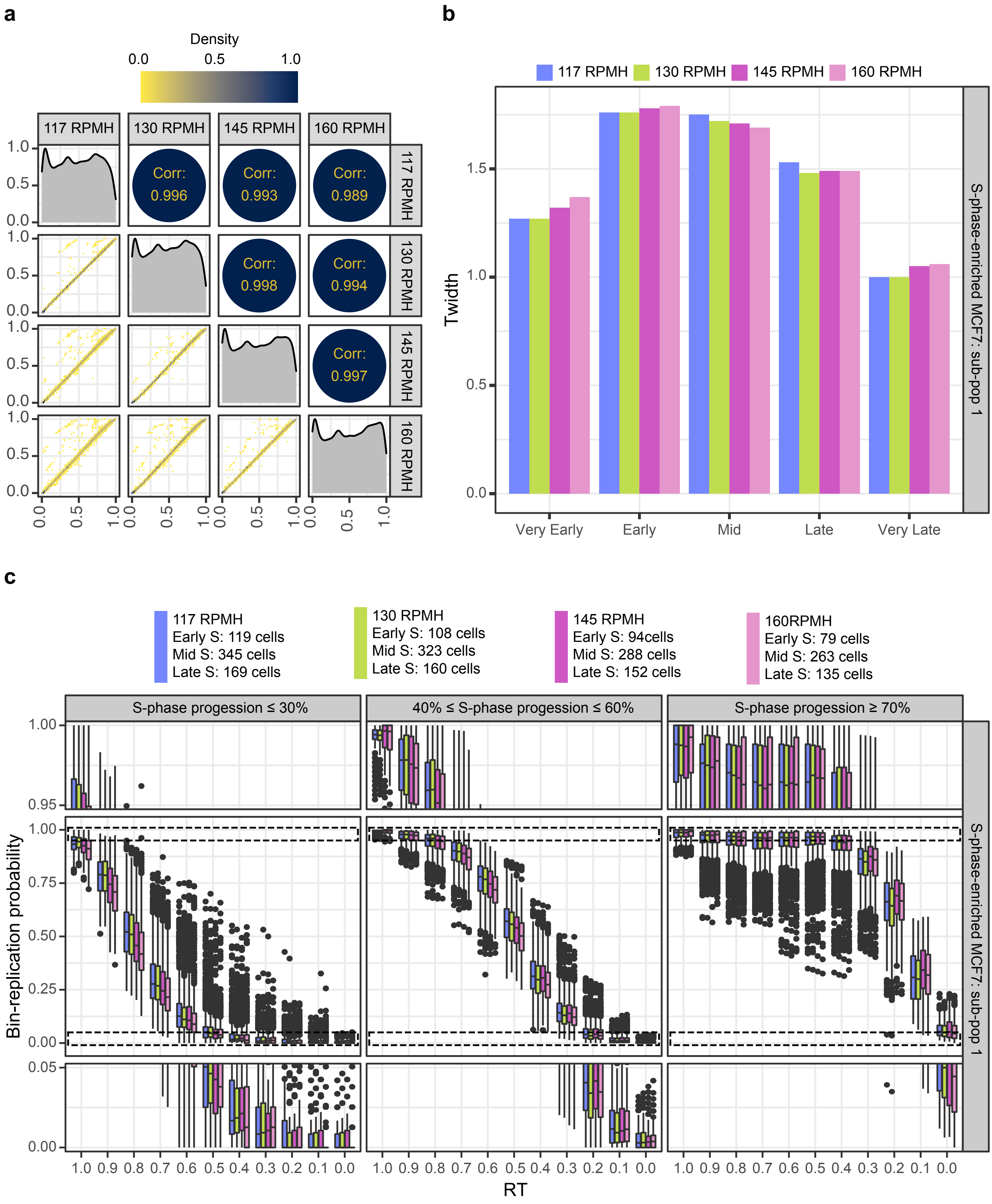

RT	1.0	0.9	0.8	0.7	0.6	0.5	0.4	0.3	0.2	0.1	0.0
117 RPMH	532	1370	1400	1455	1287	1292	1324	1303	1205	1240	1178
130 RPMH	475	1164	1401	1505	1342	1332	1442	1265	1240	1171	1248
145 RPMH	777	1449	1422	1392	1230	1237	1265	1284	1136	1102	1290
160 RPMH	728	1418	1372	1357	1268	1212	1223	1306	1154	1135	1411

Supplementary Figure 6. Key results are confirmed by using different thresholds for the minimum numbers of reads per cell. a Pairwise comparison of the pseudo-bulk RTs in the S-phase enriched MCF7 cells (sub-population 1) calculated with different Reads Per Mb per Haplotype (RPMH). **b** T_{width} values calculated in the indicated five RT categories based on the pseudo-bulk RT values obtained for the S-phase enriched MCF7 cells (sub-population 1) using different RPMH. **c** Boxplots showing the probability of a bin to be replicated (y-axis) in function of its pseudo-bulk RT (x-axis, 1 is early and 0 is late) in the S-phase enriched MCF7 cell sub-population 1 at different S-phase stages using different RPMH thresholds. In the boxplots, bounds of box: 25th and 75th percentiles; centre line: median; lower (upper) whisker: lower (upper) bound - (+) 1.5 x IQR. The numbers of genomic bins per RT category (the same for all three panels) are reported in the table below the plot.
