# Supplementary material for: Kronos scRT: a uniform framework for single-cell replication timing analysis
Source: Nat Commun. 2022 Apr 28;13:2329. doi: 10.1038/s41467-022-30043-x (PMC9050662; doi:10.1038/s41467-022-30043-x)
Supplement: Supplementary file 3 — Reporting Summary [file 41467_2022_30043_MOESM3_ESM.pdf]

Corresponding author(s): Chun-Long CHEN

Last updated by author(s): Mar 31, 2022

Reporting Summary

Nature Portfolio wishes to improve the reproducibility of the work that we publish. This form provides structure for consistency and transparency in reporting. For further information on Nature Portfolio policies, see our Editorial Policies and the Editorial Policy Checklist.

Statistics

For all statistical analyses, confirm that the following items are present in the figure legend, table legend, main text, or Methods section.

n/a	Confirmed	
☐	☒	The exact sample size (*n*) for each experimental group/condition, given as a discrete number and unit of measurement
☒	☐	A statement on whether measurements were taken from distinct samples or whether the same sample was measured repeatedly
☐	☒	The statistical test(s) used AND whether they are one- or two-sided<br>*Only common tests should be described solely by name; describe more complex techniques in the Methods section.*
☒	☐	A description of all covariates tested
☐	☒	A description of any assumptions or corrections, such as tests of normality and adjustment for multiple comparisons
☐	☒	A full description of the statistical parameters including central tendency (e.g. means) or other basic estimates (e.g. regression coefficient) AND variation (e.g. standard deviation) or associated estimates of uncertainty (e.g. confidence intervals)
☐	☒	For null hypothesis testing, the test statistic (e.g. *F*, *t*, *r*) with confidence intervals, effect sizes, degrees of freedom and *P* value noted<br>*Give P values as exact values whenever suitable.*
☒	☐	For Bayesian analysis, information on the choice of priors and Markov chain Monte Carlo settings
☒	☐	For hierarchical and complex designs, identification of the appropriate level for tests and full reporting of outcomes
☐	☒	Estimates of effect sizes (e.g. Cohen's *d*, Pearson's *r*), indicating how they were calculated

Our web collection on statistics for biologists contains articles on many of the points above.

Software and code

Policy information about availability of computer code

Data collection	MetaSystems: Metafer4, version 3.13.5 CM<br>Deltavision Core system (Applied Precision): Softworx, version 7.1.0
Data analysis	Cell Ranger DNA, version 1.0.0<br>subset-bam, version 1.0<br>Picard, version 2.6.0<br>Trim Galore, version 0.4.4<br>Kronos scRT (https://github.com/CL-CHEN-Lab/Kronos_scRT)<br>Fiji, version 2.1.0

For manuscripts utilizing custom algorithms or software that are central to the research but not yet described in published literature, software must be made available to editors and reviewers. We strongly encourage code deposition in a community repository (e.g. GitHub). See the Nature Portfolio guidelines for submitting code & software for further information.

Data

Policy information about availability of data

All manuscripts must include a data availability statement. This statement should provide the following information, where applicable:

- Accession codes, unique identifiers, or web links for publicly available datasets
- A description of any restrictions on data availability
- For clinical datasets or third party data, please ensure that the statement adheres to our policy

The mouse scWGS data were obtained from GSE108556. The mouse scHi-C data were obtained from GSE94489. The bulk RT data were obtained from GSM923442

for MCF7 cells, GSM923449 for HeLa cells, GSM923451 for GM12878 cells (B-lymphoblastoid cell line) and GSE108556 for mESC and NE-7d cells. The mm10 blacklist was obtained from https://github.com/Boyle-Lab/Blacklist. The raw and processed data generated in the current study were deposited in Gene Expression Omnibus (GEO) under accession number GSE186173.

Field-specific reporting

Please select the one below that is the best fit for your research. If you are not sure, read the appropriate sections before making your selection.

☒ Life sciences ☐ Behavioural & social sciences ☐ Ecological, evolutionary & environmental sciences

For a reference copy of the document with all sections, see nature.com/documents/nr-reporting-summary-flat.pdf

Life sciences study design

All studies must disclose on these points even when the disclosure is negative.

Sample size	Sample size consist in the total number of available cells or the number of bins as indicated in the figures and figure legends.
Data exclusions	Single cell data were excluded based on thresholds estimated through down-sampling of cells with relatively high coverage. The minimum number of reads required for CNV calling depends on the experimental settings and the procedure used to create the scWGS libraries. Therefore, for each dataset, it is important to select G1/G2- and S-phase cells with relatively high coverage to determine by down-sampling the robustness of CNV detection and the minimum number of reads required for correct CNV calling. For each dataset, from the down-sampling values, we selected a coverage threshold that did not allow the ploidy of 75% of S-phase cells to deviate more than 5% from the original ploidy estimation (see Supplementary Table2 for the cutoff used for each dataset). For the G1/G2 variability estimation (Figure S5c), to guarantee that the selection only contains cells out of S phase, only cells of MCF7 cell sub-population 1 with a mean ploidy included in the interquartile of the population were selected.
Replication	Two independent samples for Jeff and HeLa cell lines were produced. The individual analyses of these samples shows a good concordance (data not shown). In order to increase the statistical power, data of both replicates of each cell type were combined. The main conclusions of this paper are supported by the concordant results obtained in different cell types from both human and mouse. Moreover we repeated the analysis on one dataset using different thresholds and obtaining similar results. For the MCF7 cell FISH experiment we performed two independent experiments.
Randomization	We do not perform comparisons between experimental groups, therefore randomization is not relevant.
Blinding	Since there are no comparison between experimental groups we and most of the analysis are quantitative matrix we do not need blinding.

Reporting for specific materials, systems and methods

We require information from authors about some types of materials, experimental systems and methods used in many studies. Here, indicate whether each material, system or method listed is relevant to your study. If you are not sure if a list item applies to your research, read the appropriate section before selecting a response.

Materials & experimental systems

n/a	Involved in the study
☒	☐ Antibodies
☐	☒ Eukaryotic cell lines
☒	☐ Palaeontology and archaeology
☒	☐ Animals and other organisms
☒	☐ Human research participants
☒	☐ Clinical data
☒	☐ Dual use research of concern

Methods

n/a	Involved in the study
☒	☐ ChIP-seq
☒	☐ Flow cytometry
☒	☐ MRI-based neuroimaging

Eukaryotic cell lines

Policy information about cell lines

Cell line source(s)	MCF7 cells were provided by Almouzni's lab at the Institut Curie in Paris, France (obtained form ATCC). JEFF Cells were provided by Debatisse's lab at the Institut de Cancérologie Gustave Roussy in Villejuif, France. HeLa S3 cells were ordered from ATCC.
Authentication	None of the cell lines was authenticated.
Mycoplasma contamination	We routinely tested for mycoplasma with negative results.

Commonly misidentified lines<br>(See ICLAC register)	None of the used cell lines belongs to the list of commonly misidentified lines.
